# Cone Beam Computed Tomography (CBCT) Prevalence and Morphometry of Foramen Vesalius: A Systematic Review, Meta-Analysis, and Update of the Literature

**DOI:** 10.3390/jcm15062195

**Published:** 2026-03-13

**Authors:** Abdullah Hasan A. Alshehri, Anwar Abdullah Alsaeed, Hajer Saeed Al-serhani, Hassan Ahmed Assiri, Abdullah Alqarni, Saeed Alassiri, Mohammad Alamri, Sonia Egido-Moreno, José López-López

**Affiliations:** 1 Department of Prosthodontics, College of Dentistry, King Khalid University, Abha 61421, Saudi Arabia; abhalshehri@kku.edu.sa; 2 Internship Program, College of Dentistry, King Khalid University, Abha 61421, Saudi Arabia; 3 Department of Diagnostic Sciences and Oral Biology and Periodontology, College of Dentistry, King Khalid University, Abha 61421, Saudi Arabia; 4 Department of Restorative Dental Sciences, College of Dentistry, King Khalid University, Abha 61421, Saudi Arabia; 5 Department of Odontostomatology, Faculty of Medicine and Health Sciences, School of Dentistry, University Campus of Bellvitge, University of Barcelona, Barcelona Dental Hospital [HOUB], 08970 Barcelona, Spain18575jll@gmail.com (J.L.-L.)

**Keywords:** cone-beam computed tomography, CBCT, foramen of Vesalius, morphometry, prevalence

## Abstract

**Background**: The foramen of Vesalius (FV; also known as the sphenoidal emissary foramen (SEF) or foramen venosum) is an inconstant skull-base foramen located near the foramen ovale. Its recognition may be relevant to percutaneous trigeminal procedures. **Methods**: This systematic review was registered in INPLASY (INOLASY2025100037; 11 October 2025) and conducted in accordance with PRISMA 2020. We searched PubMed, Scopus, and Web of Science from inception until December 2025 for English-language studies reporting the prevalence and/or morphometry of the foramen of Vesalius using cone-beam computed tomography (CBCT). Five reviewers screened and extracted data; prevalence studies were assessed for risk of bias using the Joanna Briggs Institute checklist. A random-effects meta-analysis of logit-transformed proportions was applied when ≥3 studies reported comparable prevalence outcomes. **Results**: Five retrospective CBCT studies (*n* = 1567) met the inclusion criteria. The prevalence of FV ranged from 28.1% (89/317; 95% CI 23.4–33.3) to 73.1% (190/260; 95% CI 67.4–78.1) throughout the cohorts. The total prevalence was 50.6% (95% CI 36.1–65.1), with significant variability (I^2^ = 96.8%) and a broad 95% prediction interval (19.5–81.3). The documented FV–FO distances were typically a few millimeters (about 2–5 mm), whereas the FV–foramen spinosum (FS) distances varied from approximately 11 to 14 mm, contingent upon the cohort and measuring technique employed. **Conclusions**: FV is frequently observable on CBCT when the skull base is within the field of view; nevertheless, current prevalence estimates lack precision because of the limited number of five retrospective investigations, which are inconsistent and clinic-based. Standardized definitions and reporting for CBCT, together with population-based cohorts, are crucial for improving clinically applicable prevalence and morphometric reference data.

## 1. Introduction

The foramen of Vesalius, also known as the sphenoidal emissary foramen, is a small but significant anatomical structure located near the vidian canal and anterior to the foramen ovale. It typically exhibits bilateral symmetry when present, though varying in its size and presence. This foramen contains an emissary vein that facilitates blood flow between the cavernous sinus and the pterygoid venous plexus. It could serve as a path for infections or blood clots to migrate between the interior and exterior of the skull [1,2]. Clinically, FV is significant due to its proximity to the foramen ovale. This relationship is particularly relevant during procedures targeting the foramen ovale, including percutaneous approaches for trigeminal neuralgia, and during skull-base and maxillofacial interventions in the middle cranial fossa, where unrecognised variant foramina and emissary venous connections may increase the risk of venous injury or procedural misdirection. In the neurosurgical literature, failure to accurately cannulate the foramen ovale has been associated with severe adverse events, and mistaken cannulation of a nearby sphenoidal emissary foramen has been specifically described as a mechanism for cavernous sinus injury and intracranial haemorrhagic complications [3].

The foramen linking the cavernous sinus and the pterygoid venous plexus was first described by the anatomist Andreas Vesalius. This venous connection is possibly part of the intracranial anastomosis that connects the venous systems of the brain and face. Research on desiccated specimens indicates that FV is found in 5–80% of skulls [4,5]. A significant clinical relevance of FV is its proximity to the foramen ovale during percutaneous trigeminal interventions, such as radiofrequency rhizotomy, glycerol rhizotomy, or balloon compression, where cannulation is aimed at the foramen ovale. In some cases, an additional FV may be accidentally cannulated, which could increase the risk of venous injury or complications within the skull [6,7]. CBCT provides clear three-dimensional images of the mouth and face, enabling examination from various angles and a better understanding of their appearance. Traditional procedures, such as panoramic and periapical X-rays, do not always effectively display the complex three-dimensional anatomy of the mouth and teeth due to superimposition and geometric distortion. Consequently, significant clinical findings, including periapical lesions, root morphology, and the relationship between teeth and alveolar bone, may be obscured [6,8]. A study conducted on 19 December 2018, at Eskisehir Osmangazi University in Turkey evaluated the prevalence of the foramen meningo-orbitale, craniopharyngeal canals, canaliculus innominatus, FV, palatovaginal canals, and canalis basalis medianus by age and gender in a cohort of 350 individuals (200 females and 150 males) who underwent CBCT, with a mean age of 15. The findings indicated that FV was present in 145 out of 350 samples (41.1%), with the predominant age group affected being 22 to 30. Females demonstrated a greater prevalence of FV (42.5%) than males [4,9]. A 2007 examination of a dried adult skull at Istanbul University in Turkey found that 87 of the 347 samples (25.1%) had bilateral foramina vascularia, while 69 (19.9%) had unilateral foramina vascularia. Of the 69 skulls, 33 had unilateral FV on the right side, and another 36 had unilateral FV on the left. There was no FV on either side of the other 191 samples (55%) [10,11]. The anatomical and radiological literature employs many terminologies for the foramen of Vesalius. It is also referred to as the sphenoidal emissary foramen or foramen venosum. The stated prevalence differs markedly between cadaveric and conventional CT examinations. The detectability of CBCT is likely influenced by acquisition parameters such as voxel size and field of view and operational imaging requirements, including minimal visibility on successive slices, multiplanar confirmation, and patency or continuity standards. This systematic review aimed to address the CBCT-derived evidence regarding the prevalence, morphometries, and laterality of FV.

## 2. Methodology

This systematic review protocol was registered in INPLASY under the registration number (INPLASY2025100037) on 11 October 2025 and approved by the KKUCOD research ethics committee (IRB/REG/2025/9). The review adheres to PRISMA 2020 guidelines. The review question employed a prevalence-focused framework (Population/Context–Concept–Outcome). Population/Context: individuals undergoing CBCT examinations in which the skull base is adequately visualized. Concept: FV, referred to as the SEF/foramen venosum, was identified on CBCT using each study’s radiological definition. Outcome measures include FV prevalence (overall and by side when reported) and FV morphometry, such as shape, diameter, and distances to adjacent foramina when available. Due to the expected clinical and methodological heterogeneity, findings were summarized narratively; where three or more studies reported comparable prevalence outcomes, a random-effects meta-analysis of proportions (logit-transformed) was performed, and pooled estimates were interpreted cautiously in view of heterogeneity.

The study addressed the following research question:

What are the prevalence and morphometric characteristics of the foramen of Vesalius (FV), as assessed by cone-beam computed tomography (CBCT)?

### 2.1. Inclusion and Exclusion Criteria

The inclusion criteria were original studies that described the prevalence and characteristics of the foramen using CBCT and studies published in English. We excluded reports, reviews, and studies in other languages.

### 2.2. Search Strategy

PubMed, Scopus, and Web of Science were searched in December 2025, using combinations of CBCT terms and FV/SEF terms with database-appropriate syntax:

PubMed: ((“cone-beam computed tomography” OR CBCT) AND (“foramen of Vesalius” OR “foramen Vesalius” OR “sphenoidal emissary foramen” OR “foramen venosum”));

Scopus: TITLE-ABS-KEY ((“cone beam computed tomography” OR CBCT) AND (“foramen Vesalius” OR “foramen of Vesalius” OR “sphenoidal emissary foramen” OR “foramen venosum”));

Web of Science: TS = ((“cone beam computed tomography” OR CBCT) AND (“foramen Vesalius” OR “foramen of Vesalius” OR “sphenoidal emissary foramen” OR “foramen venosum”)).

Additionally, the reference lists of included studies were screened for eligible reports.

### 2.3. Study Retrieval

**Figure 1:**
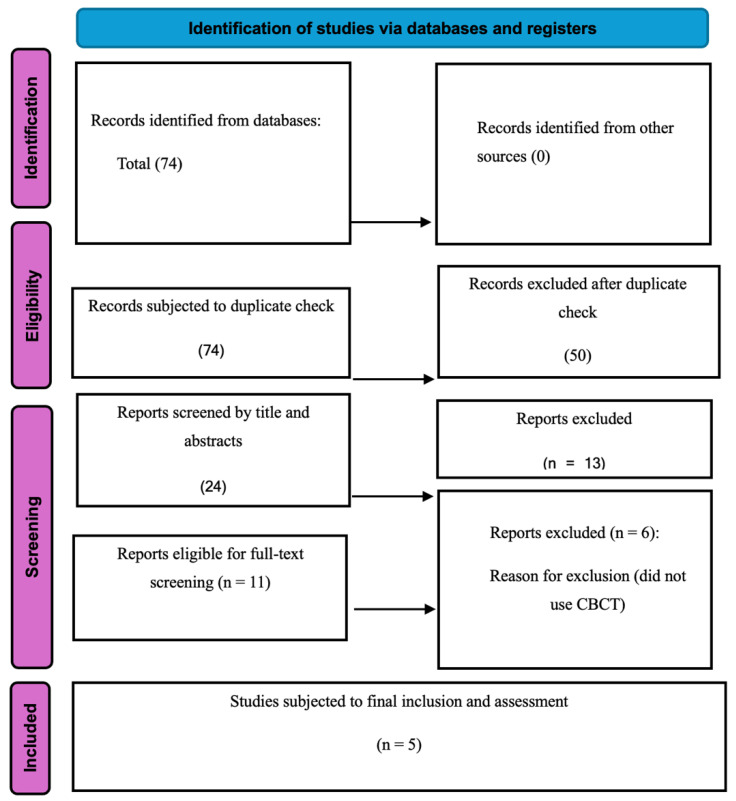
PRISMA flow chart of the systematic review.

Seventy-four records were identified from the database search. After removing duplicates (*n* = 50), the remaining 24 were screened by title and abstract. Thirteen records were excluded. Eleven full-text articles were assessed for eligibility; six were excluded because CBCT was not used for FV identification. Five studies met the inclusion criteria and were included in the qualitative synthesis (total *n* = 1567). The PRISMA flow diagram summarizes the study selection process (Figure 1).

### 2.4. Data Collection

The studies included were subjected to data extraction according to the pre-identified working table (Table 1). Five authors divided the included articles for extraction. In cases of uncertainty, the authors consulted for a consensus. Data were extracted based on criteria including author and year, sample, objective, CBCT protocol, outcomes (FV identification), and methodological strengths.

### 2.5. Risk of Bias Assessment

All five authors independently appraised the included studies using the Joanna Briggs Institute (JBI) Critical Appraisal Checklist for Prevalence Studies. Each item was judged as Yes/No/Unclear/Not applicable. Any disagreements were resolved through consensus. An overall risk-of-bias judgement (low/moderate/high) was assigned based on the pattern and seriousness of methodological limitations (e.g., sampling frame, sampling method, measurement reliability), rather than by applying a numerical cut-off score (Table 2).

### 2.6. Meta-Analysis

When three or more studies reported comparable prevalence outcomes, a meta-analysis of proportions was performed. For each study, prevalence was defined as the proportion of CBCT scans demonstrating the presence of FV (unilateral or bilateral) among all eligible scans. Pooled prevalence was calculated using a random-effects model (DerSimonian–Laird) on logit-transformed proportions, with back-transformation to prevalence. Statistical heterogeneity was assessed using Cochran’s Q and the I^2^ statistic, and a 95% prediction interval was calculated to reflect between-study variability. Meta-analysis and forest plots were generated in Stata 17.0 (StataCorp, College Station, TX, USA) using generic inverse-variance methods. Individual study prevalence estimates (p) were transformed using the logit transformation, logit(p) = ln[p/(1 − p)]. Standard errors (SE) were derived from binomial variance as SE = √ (1/e + 1/(n − e)), where (e) is the number of CBCT scans with FV and (n) the study sample size. When studies reported prevalence percentages without raw counts, SE was computed using SE = √(1/(n·p) + 1/(n·(1 − p))). Pooled estimates were back-transformed to prevalence using the inverse-logit function for interpretation and display. Statistical heterogeneity was assessed through the Chi-square test and I^2^ statistic. For descriptive reporting, we calculated Wilson 95% confidence intervals for each individual study prevalence estimate; when a study reported prevalence solely as percentages, event counts were estimated as p × n to facilitate confidence interval estimation and meta-analysis.

## 3. Results

### 3.1. Study Selection

Seventy-four records were identified from the database search. All records were imported into Rayyan^®^ with reference management and de-duplication. After duplicates (*n* = 50) were removed, the titles and abstracts of the remaining records (*n* = 24) were individually screened by five reviewers using the established eligibility criteria. Eleven full-text articles were assessed for eligibility, and six did not use CBCT for FV identification. Thus, five studies met the criteria and were included in the qualitative synthesis (total *n* = 1567). The PRISMA flow diagram summarizes the study selection process (Figure 1).

### 3.2. Data Extraction

The studies included for final analysis underwent data extraction before risk-of-bias assessment. Extracted items included study design/setting, sample characteristics, CBCT acquisition/analysis details, FV definition/reading rules, prevalence (including laterality when available), and reported morphometry (Table 1). The reported CBCT scans were obtained from university and hospital radiology archives, corresponding to clinical indications. Sample sizes ranged from 140 to 500, with age distributions revealing a wide spectrum from pediatric and young adult cohorts to elderly persons. The protocols for CBCT acquisition and assessment were varied, illustrating discrepancies in kVp/mA settings, voxel dimensions, and field of view when specified, as well as variations in radiological case definitions.

### 3.3. Risk of Bias Assessment

The included studies were assessed for risk of bias using the Joanna Briggs Institute (JBI) checklist for prevalence studies. Overall, the five included studies demonstrated predominantly moderate risk of bias, largely driven by retrospective designs, clinic-based or convenience sampling frames, and incomplete reporting of sampling strategies and prevalence precision, despite generally clear CBCT-based definitions and structured image evaluation methods (Table 2).

### 3.4. Meta-Analysis

**Figure 2:**
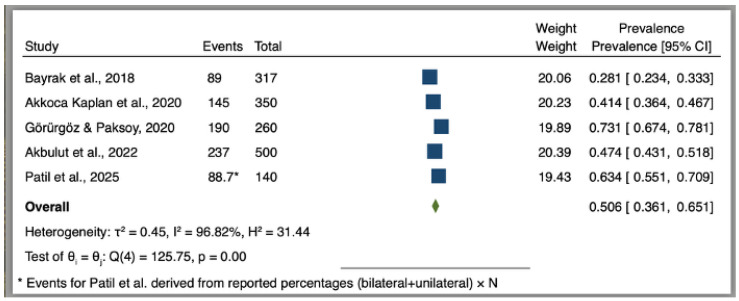
Forest plot of the prevalence of FV identified on CBCT. Random-effects meta-analysis (DerSimonian–Laird) of logit-transformed proportions performed in Stata 17.0, with inverse-logit back-transformation to prevalence for display. Individual study prevalence estimates are shown with Wilson 95% confidence intervals. Heterogeneity: τ^2^ = 0.45; Q (4) = 125.75; I^2^ = 96.82%; *p* < 0.0001. Events for Patil et al. were derived from reported percentages (bilateral + unilateral) × N.

The prevalence of FV on CBCT showed considerable diversity throughout the five retrospective studies (*n* = 1567), with rates varying from 28.1% to 73.1%. According to Wilson’s 95% confidence interval, the study-level prevalence estimates were as follows: 28.1% (89/317; 23.4–33.3) in Bayrak et al.; 41.4% (145/350; 36.4–46.7) in Akkoca Kaplan et al.; 47.4% (237/500; 43.1–51.8) in Akbulut et al.; 63.4% (approximately 89/140; 55.3–71.1) in Patil et al. (event count approximated from reported percentages); and 73.1% (190/260; 67.4–78.1) in Görürgöz & Paksoy. In Stata 17.0, logit-transformed proportions were integrated using an inverse-variance, DerSimonian–Laird random-effects model and subsequently converted back to prevalence through the inverse-logit approach for presentation. The total prevalence of FV was 50.6% (95% CI 36.1–65.1%). Significant heterogeneity was observed among the studies (τ^2^ = 0.45; Q (4) = 125.75; *p* < 0.0001; I^2^ = 96.82%), with a broad 95% prediction interval (19.5–81.3%). The discrepancies arose from variations in sampling frames, age distributions, operational definitions of FV, reading criteria, and CBCT acquisition parameters and fields of view. Our exploratory leave-one-out sensitivity analysis revealed that the pooled prevalence estimates varied between 44.5% and 56.6%. This indicates that the imprecision and heterogeneity remained unchanged despite the exclusion of one study. Thus, the combined value should be viewed as an average across several clinical situations rather than a solitary population prevalence, maintaining emphasis on study-level estimates and clinically relevant morphometry (Figure 2).

## 4. Discussion

In this systematic review of CBCT-based evidence, FV prevalence showed substantial variability across five included studies (*n* = 1567). Many cohorts revealed intermediate prevalence, and one recent CBCT study documented FV more commonly as bilateral than unilateral (Table 1). This spread is plausibly explained by methodological heterogeneity (age ranges, indications for CBCT, sampling frame, CBCT acquisition/voxel parameters, and operational definitions of FV, such as corticated margins and visibility on consecutive sections) [13]. Therefore, a single pooled prevalence without careful handling of heterogeneity would be difficult to interpret clinically; the findings are best summarized using narrative synthesis and study-level estimates alongside morphometric measures relevant to surgical planning. Clinical relevance: FV is, therefore, not rare on CBCT, and CBCT can provide actionable morphometric information. Several included studies reported FV–foramen ovale distances of only a few millimeters, supporting the anatomic premise that FV may be encountered close to, or confluent with, the foramen ovale. Where skull-base anatomy is included in a CBCT field of view, identifying and explicitly reporting FV (presence, laterality, and relationship to the foramen ovale) may assist clinicians planning skull-base interventions or maxillofacial procedures, and may reduce the chance of inadvertent cannulation of adjacent foramina during transovale approaches described in the neurosurgical literature [3]. The included studies point out that FV is not a rare find in case the CBCT imaging protocol covers the skull base. However, the prevalence estimates differ meaningfully across different samples (Table 1). These findings align with the median range of anatomical descriptions from dry-skull series, which have traditionally demonstrated significant variability in prevalence (5–80%) [2]. The 2007 Istanbul University dry-skull study revealed that 45% of the population had FV (25.1% bilaterally and 19.9% unilaterally), while 55% did not. This percentage is slightly higher than the pooled proportion based on CBCT, although it aligns with the assumption that FV is frequent in the general population [6]. The imaging-based rates from CBCT cohorts and the cadaveric rates from historical anatomy underscore genuine population variability, possibly influenced by ethnicity, definitions (e.g., corticalized foramen versus canaliculi), and study design, rather than a shortcoming of CBCT in depicting the foramen. Regarding the laterality of the FV, our study indicated that both studies found both unilateral and bilateral FV on CBCT [8,10]. In the study reported by Bayrak et al. [8], the unilateral FV was slightly more frequent on the right, and the left FV is reported to occur near the midline. Nevertheless, there was no clear consistency of laterality across the included studies. In the Istanbul dry-skull series, unilateral presence was nearly symmetric (right 33 vs. left 36), indicating a lack of strong side dominance across methods [10]. This concordance in laterality patterns between CBCT and dry-skull data supports the anatomical plausibility and external consistency of CBCT observations [6]. CBCT imaging enables the assessment of FV using reproducible and explicit radiological criteria, most commonly a corticated foramen with a central radiolucency visible on consecutive sections and confirmed on multiplanar reconstructions. Additionally, CBCT allows direct measurements of clinically relevant morphometries and of the spatial resolution between the FV and adjacent foramina. Across the CBCT samples, the FV–foramen ovale separation was typically only a few millimeters, while the FV–foramen spinosum distance was in the order of centimeters. This underscores the close spatial relationship between the FV and the neighboring foramina that are relevant to the skull base interventions.

From a practical perspective, when the skull base is included within the CBCT field of view, FV assessment can be incorporated into routine interpretation by confirming the foramen on multiplanar reconstructions and identifying its presence, laterality/duplication, and spatial relationship to the foramen ovale. Explicit reporting of a patent FV adjacent to the foramen ovale, especially when the separation is only a few millimeters, may aid clinicians in anticipating a potential alternative cannulation pathway and refine image-guided trajectories, thereby reducing the likelihood of procedural misdirection and venous injury. On the other hand, ‘non-visualization’ of the FV should be interpreted in the light of technical constraints (e.g., limited field of view or reduced spatial resolution) since small foramina may fall below the detection threshold under inadequate imaging conditions.

Limitations and future research: This review is limited by the small number of eligible CBCT studies (*n* = 5) and the predominance of retrospective, clinic-based sampling frames, which may introduce selection bias and restrict generalizability to broader populations. Operational imaging definitions and reporting practices varied across studies, and several papers did not fully report CBCT acquisition parameters (e.g., voxel size, field of view, exposure settings) or reproducibility metrics (intra-/inter-observer agreement), which may contribute to the misclassification of small foramina and to the substantial between-study heterogeneity observed. Additionally, morphometric endpoints were inconsistently reported, preventing robust quantitative pooling beyond overall prevalence, and publication bias could not be reliably assessed with the limited number of studies. Future research should prioritize multicenter, prospectively designed, or population-based CBCT cohorts with explicit sampling (consecutive or random), standardized FV imaging definitions (multiplanar confirmation and a minimum visibility criterion), complete reporting of CBCT parameters, and reproducibility testing. Studies should report prevalence with 95% confidence intervals and stratify estimates by age, sex, and population/ethnicity; where feasible, validation against an anatomical or cross-sectional imaging reference standard in a subset would help confirm case definitions and refine clinically actionable morphometric thresholds (e.g., FV proximity to the foramen ovale).

## 5. Conclusions

In five retrospective, clinic-based CBCT studies, FV was identified in a substantial proportion of scans (28.1–73.1%). The aggregated prevalence was 50.6%; nevertheless, the groups exhibited significant differences, indicating that this figure should be interpreted as an average among diverse groups rather than a singular population prevalence. Due to the proximity of FV to the foramen ovale, CBCT visualization of the skull base may facilitate safer procedural planning. Future population-based CBCT investigations utilizing established imaging definitions, thorough reporting of acquisition parameters, and reproducible measurement techniques are crucial for generating more precise and clinically relevant prevalence and morphometric reference data.

## Figures and Tables

**Table 1:** Data extraction of included studies according to predefined criteria.

Study (Year)	Design/Setting	Sample	CBCT Protocol/Software	FV Definition & Reading Rule	Key Outcomes (Prevalence & Morphometry)	Notes/Strengths
Bayrak et al. (2018) [8]	Retrospective clinical CBCT series (university outpatient).	*n* = 317 (189 F/128 M); age 13–58 (mean 18.34 ± 6.6). Excluded: bone disease/related drugs, trauma/asymmetry, congenital disease, prior surgery, tumor; inadequate scans excluded.	i-CAT 3D; 120 kVp, 5 mA; FOV 16 × 13 cm; axial + 3D reconstructions.	FV is defined as a round/oval opening with a sclerotic rim and central lucency visible on ≥2 adjacent slices; evaluated on CBCT with multiplanar/3D confirmation.	Prevalence: 89/317 (28.1%; 95% CI 23.4–33.3); unilateral 21.1% (R 36, L 31); bilateral 6.9%. Shape: oval 68.5%, round 25.2%, irregular 6.3%. Max diameter: R 2.66 mm, L 2.82 mm (NS). Distances: FV–FO R 2.31 mm/L 2.21 mm; FV–FS ≈ 11.3 mm; FV–midline R 19.57 mm/L 15.80 mm (L closer).	Two observers with repeated measures, high intra-/inter-observer agreement, clear exclusions and statistics, and IRB reported.
Akkoca Kaplan et al. (2020) [10]	Retrospective radiological database (single center).	*n* = 350 (200 F/150 M); age 6–30 (mean 15.1 ± 4.08). Excluded: syndromes, neurological disorders, and prior procedures around interest.	Planmeca ProMax 3D Mid; 94 kVp, 14 mA; 360° rotation, 27 s; voxel 0.600; multiplanar (axial/coronal/sagittal).	FV delineated radiologically between FO and foramen rotundum, confirmed in axial/coronal/sagittal planes (MPR validation).	Prevalence: 145/350 (41.4%; 95% CI 36.4–46.7). Age strata: 6–15 years 42.8%, 16–21 years 30.2%, 22–30 years 66.7%. Sex: F 42.5%, M 40.0% (no significant sex effect).	Examiner consistency was assessed (10 cases re-read after 2 weeks; r = 0.85–0.96); a standardized protocol was used; and age/sex-stratified reporting was done.
Görürgöz & Paksoy (2020) [12]	Retrospective CBCT archive study.	269 CBCT scans evaluated; 9 excluded (duplicate foramina) → *n* = 260 analysed.	Planmeca ProMax 3D Max; 96 kVp, 5.6–12 mA; 9–15 s scan time; FOV 13 × 9 cm or 23 × 16 cm; voxel 200 or 400 µm; reconstructed at 0.5 mm slice interval/thickness, evaluation using Romexis 3.7 (Planmeca) with multiplanar (sagittal/coronal/axial) review.	FV identified by demonstrating continuity through the infratemporal fossa to the middle cranial fossa; blind-ending canals were recorded as absent. FV morphology, morphometry, laterality, and occurrence were reported.	Prevalence: 190/260 (73.1%; 95% CI 67.4–78.1). Laterality (per-side reporting): right 148/260 (56.9%), left 152/260 (58.5%). Unilateral 80/260 (30.8%); bilateral 110/260 (42.3%). Max diameter: 1.75 ± 1.27 mm. Shape: most commonly oval (R 45.9%, L 40.8%).	Reports laterality and morphometry alongside prevalence; large sample.
Akbulut et al. (2022) [13]	Clinic-based retrospective cohort (2012–2020).	*n* = 500 CBCT scans.	i-CAT Model 17–19; voxel 0.3 mm; 360° rotation; 120 kV, 5.0 mA, 4.8 s; FOV 9–13 × 16 mm; analysis with Invivo 5.2.	FV defined radiographically and required visibility on ≥2 consecutive sections (per extracted table).	Prevalence: 237/500 (47.4%; 95% CI 43.1–51.8). Among FV-positive scans: unilateral 70/237 (29.5%), bilateral 167/237 (70.5%). Diameter: 1.07 ± 0.50 mm. Distances: FV–FO 3.29 ± 2.11 mm; FV–FS 12.44 ± 2.85 mm; FV–midline 19.20 ± 3.23 mm.	Standardized acquisition; explicit definition, and comprehensive morphometry with dispersion measures.
Patil et al. (2025) [14]	Retrospective CBCT study (dental hospital setting).	*n* = 140 scans (68 M/72 F); age 11–70 years; diagnostic-quality skull-base visualization required.	Image analysis in Planmeca Romexis 5.3; the CBCT unit and acquisition parameters (e.g., kVp, mA, voxel size, FOV) were not reported in the primary study.	FV assessed in the expected anatomical location; evaluation was performed in axial section using 3D-rendered images in Romexis. A minimum visibility criterion (e.g., consecutive slices) was not explicitly documented.	Laterality/prevalence categories: bilateral FV 44.64%, unilateral FV 18.72%, absent FV 37.44%. (FV present ≈ 89/140, 63.4%; 95% CI 55.3–71.1; counts approximated from reported percentages). FV–FO and FV–FS distances reported by sex and side (see primary paper for full numeric table).	Clinically relevant morphometrics (distances to FO/FS) stratified by sex/side; explicit inclusion/exclusion criteria. Acquisition parameters and an explicit FV visibility threshold were not fully reported, limiting cross-study comparability.

**Table 2:** Bias risk assessment using the JBI checklist tool.

JBI Checklist Item	Bayrak et al., 2018 [8]		Akkoca Kaplan et al., 2020 [10]		Görürgöz & Paksoy, 2020 [12]		Akbulut et al., 2022 [13]		Patil et al., 2025 [14]	
	Score	Interpretation	Score	Interpretation	Score	Interpretation	Score	Interpretation	Score	Interpretation
1. Was the sample frame appropriate to address the target population?	No	Clinic-based CBCTs; not population-based; age-skewed (~18 years).	No	Clinic radiology database (ages 6–30); not population-based.	No	Clinically indicated CBCT archive; not population-based.	No	Single-centre clinic CBCT archive; not population-based.	No	Single-centre retrospective dental hospital CBCTs; convenience sample.
2. Were study participants sampled appropriately?	Unclear	Sampling method (random vs. consecutive) not described.	Yes	Random selection from the radiology database reported.	Yes	CBCT scans randomly selected from digital archive by radiologist.	Unclear	Sampling strategy (random/consecutive) not stated.	No	Convenience sampling explicitly reported.
3. Was the sample size adequate?	Unclear	No a priori sample-size/precision calculation reported.	Unclear	No a priori sample-size/precision calculation reported.	Unclear	No a priori sample-size/precision calculation reported.	Unclear	No a priori sample-size/precision calculation reported (despite *n* = 500).	Yes	Sample size estimated (absolute precision 5%, 95% confidence).
4. Were the study subjects and the setting described in detail?	Yes	Eligibility, demographics, CBCT parameters, and case definition described.	Yes	Demographics, imaging protocol, eligibility, and FV definition described.	Yes	Setting, inclusion/exclusion, demographics, and CBCT parameters detailed.	Yes	Study period, setting, eligibility criteria, and demographics described.	Yes	Age/sex distribution and inclusion/exclusion criteria reported.
5. Was the data analysis conducted with sufficient coverage of the identified sample?	Yes	Eligible scans analysed; denominators clear; image-quality exclusions noted.	Yes	All included scans analysed with clear denominators and stratified reporting.	Yes	Exclusions reported (duplicates); final denominators clear.	Yes	Full sample analysed with prevalence reporting.	Yes	Included scans analysed; prevalence stratified by sex/age.
6. Were valid methods used for the identification of the condition?	Yes	Explicit FV radiographic definition (≥2 adjacent slices) with MPR/3D confirmation.	Yes	Radiologic definition + multiplanar confirmation (axial/coronal/sagittal).	Yes	Explicit FV identification criterion (3D continuity; blind ending = absent) and MPR review.	Yes	FV defined radiographically (sclerotic borders + radiolucent centre on ≥2 sections).	Unclear	FV assessment described generally; explicit radiographic case definition not detailed.
7. Was the condition measured in a standard, reliable way for all participants?	Yes	Standardised assessment; repeated measures; inter/intra-observer reliability reported.	Yes	Single trained reader; repeat reads on subset with reported agreement.	Yes	The same radiologist assessed all scans; measurements were repeated twice and averaged.	Unclear	No explicit inter-/intra-observer reliability or calibration reported.	Unclear	No reader calibration or inter-/intra-observer reliability reported.
8. Was there appropriate statistical analysis?	Yes	Appropriate tests reported; prevalence CIs not provided.	Yes	Appropriate descriptive and inferential statistics reported.	Yes	Appropriate statistical methods described (SPSS 21.0; tests; *p* < 0.05).	Yes	Appropriate statistical analyses described (SPSS; chi-square/Fisher, etc.).	Yes	Appropriate descriptive statistics + *t*-test/chi-square reported.
9. Was the response rate adequate, and if not, was the low response rate managed appropriately?	N/A	Retrospective image database (no participant response process).	N/A	Retrospective image database (no participant response process).	N/A	Retrospective image database (no participant response process).	N/A	Retrospective image database (no participant response process).	N/A	Retrospective image database (no participant response process).
Overall risk of bias (JBI prevalence checklist)	Moderate	Main limitations: non-population frame and unclear sampling; otherwise, robust measurement.	Moderate	Main limitations: non-population frame and no sample-size justification.	Moderate	Main limitations: non-population frame and no sample-size justification.	Moderate	Limitations: non-population frame, unclear sampling, and unreported measurement reliability.	Moderate	Key concerns: convenience sampling and limited reporting of measurement reliability.

Yes = meets criterion; No = does not meet; Unclear = insufficient detail; N/A = not applicable to retrospective image datasets.

## Data Availability

The original contributions presented in this study are included in the article/Supplementary Material. Further inquiries can be directed to the corresponding author.

## Supplementary Materials

The following supporting information can be downloaded at: https://www.mdpi.com/article/10.3390/jcm15062195/s1, PRISMA 2020 Checklist, reference [15] is cited in Supplementary Materials.
